# The Administration of Anæsthetics

**Published:** 1886-05-20

**Authors:** W. Joseph Hearn

**Affiliations:** One of the Surgeons to the Hospital


### THE ADMINISTRATION OF ANÆSTHETICS.

**A Lecture delivered at the Philadelphia Hospital,**

By W. JOSEPH HEARN, M. D.,

**One of the Surgeons to the Hospital, (Reported for the College and Clinical Record.)**

GENTLEMEN : I propose to devote this hour to the subject of anæsthesia. No matter where you practice, you will all be compelled to give anæsthetics more or less frequently. In these times, when machinery of all kinds is found on every farm, accidents are of frequent occurrence, and it is important, therefore, that you should be familiar with the proper method of inducing anæsthesia. There is no subject less taught in our schools than this.

When you start in practice you should have some confidential friend whom you can get to administer the anæsthetic for you when necessary. He should be one whom you can trust. I have one friend who assists me very often in this way. I have another who administers ether for me in very difficult cases. The anæsthetizer should be a man who can give his entire attention to his duty, without trying to look at the operation. The man who persists in trying to see what is going on is not fit to give an anæsthetic. A person under ether requires constant watching. The anæsthetizer should watch the countenance, the respiration, and the pulse, and should have his entire attention directed to the patient, and not for a moment have it detracted to the surroundings. You should not give ether alone, for you do not know at what moment an accident may occur.

There are other reasons why you should not administer ether alone—especially in the case of females. Several suits for criminal assault have been brought against physicians under such circumstances. I must, however, say that, although I have been what might be called a professional anæsthetizer for fifteen years, I have never seen any erotic manifestations in patients under the influence of anæsthetics. I have taken some pains to learn whether such a thing did occur or not. In some cases I could not ascertain if such had occurred, and in others the patients denied it. Though I have not seen it, I do not deny the occurrence. I say this at this time because there is a great deal said in the books on jurisprudence about females being outraged while in the anæsthetic condition. I imagine that ether would be more apt to produce erotic manifestations than chloroform.

Every surgeon of any experience has his private anæsthetizer, and every hospital should, I think, have the same. As a rule, this duty falls upon the resident who has just graduated. This is not fair to the resident, nor is it fair to the patient or to the operator. I have never seen any accident from anæsthesia except in the hands of untrained men, and the only death which I have seen was, I am certain, the result of maladministration.

Students should have at least six months' training in the hospital, with close watching of the administration of the anæsthetic, before being allowed to give it to a patient. This is why I say that every hospital should have a professional anæsthetizer. It is claimed that, under such circumstances, the resident would never learn. He could, however, learn under the personal supervision of the anæsthetizer.

I wish, next, to say a word about anæsthetic delusions, as they may be called. Among the laity there is an idea that it is possible to render a person unconscious by simply throwing in their face a handkerchief saturated with chloroform or ether. This is impossible. It is asserted that females have been rendered unconscious in this way, and outraged. Either the female was very willing or she was so frightened that she could not resist, and this is quite probable.

Again, one frequently reads of chloroform being thrown into a room, and the house robbed while the persons were under the influence of the anæsthetic. The vapor of ether or chloroform is heavier than air, and you can imagine the immense amount that would be required to reach the height of an ordinary bedstead.

Another delusion is found when you are administering anæsthetics. The friends imagine that the patient should become unconscious the moment the ether is applied. You will constantly have to combat this idea in practice.

Many persons imagine that they talk a great deal, and tell things they do not wish, while under the influence of the anæsthetic. I have never heard a patient say anything particularly improper, although they are sometimes known to use some profanity. Many persons have a fear of being anæsthetized while asleep. This is possible in a child, with chloroform, but not with ether. In adults it would be impossible with either agent. The late Dr. Maury, one of the surgeons of this Hospital, attempted to induce anæsthesia, with chloroform, on patients who were asleep, but failed in every attempt.

I do not know of anything which I should regard as a positive contra-indication to the use of ether in a case requiring surgical operation. In a case of Bright's disease there is a little more danger than where this complication does not exist, because of the disability of the kidneys; elimination does not take place as rapidly as it should. A weak heart is no contra-indication to the administration of ether. Ether is a stimulant to the heart, and under its use the pulse improves.

I have never seen a death from ether or chloroform, although I

have seen one from the bromide of ethyl. I have only seen one case in which dangerous symptoms arose during the administration of ether. This was in the case of a man who, ten years previously, had taken chloroform and almost died. I administered ether, and the result was almost fatal.

What are the signs of danger in anæsthesia from ether? Pallor and lividity indicate failing heart and respiration. It is said that ether kills by paralysis of the respiration, but death does not always occur in that way. It may result from paralysis of the heart. Stertorous breathing is one sign of danger. The usual cause of death is an overdose of the anæsthetic. Chloroform acts differently. As small a quantity as eighteen minims of chloroform are said to have caused death.

I shall, in this connection, speak of the proper method of treatment under such circumstances. The patient is placed on the floor and the ether expelled from the lungs by pressing the sides. Artificial respiration should then be performed by carrying the arms outward and then bringing them down against the sides of the chest. This should be done regularly about eighteen times a minute. The tongue is to be drawn forward and the trachea and bronchi put on a straight line. If there should have been vomiting, and there is any suspicion of food lodged in the larynx, tracheotomy should be performed. Flagellations with a wet towel should also be practiced, in order to excite the vaso-motor nerves and cause the patient to gasp. In hospitals, a battery is usually accessible, and then the positive pole may be placed over the phrenic nerve, while the negative pole is placed over the epigastrium, and in this way contraction of the diaphragm be induced.

Occasionally spasm of the glottis occurs, the patient cannot get his breath, and becomes cyanosed. The proper plan is to remove the ether until the patient has begun to breathe properly, and then resume the anæsthetic.

A word as to the proper quantity of ether to order. If you are going simply to anæsthetize a patient in order to open an abscess or examine a fracture, four ounces should be ordered. If a more extensive operation is contemplated, eight ounces should be procured; and if a prolonged operation is expected, sixteen ounces may be needed. Whether you use ether or chloroform, it should be of the purest character. There is a form of chloroform which is very dangerous, and there is a washed chloroform, which is not so dangerous. You should get the best. I prefer Squibb's ether, because it comes in cans hermetically sealed.

In preparing the patient for the operation, see that false teeth, pins and tobacco are removed, and that there is no constriction around the waste or neck; and in this do not trust to the patient's word, but see for yourself. Ladies will deny that their corsets are tight, when it is evident that the functions of the diaphragm are interfered with.

If chloroform is to be administered, the patient should be absolutely recumbent. If ether is to be used, this is not so essential. Where there is opportunity for it, the patient should be ordered to eat no solid food previous to the operation, for the anxiety connected with the operation will interfere with digestion. This caution is especially important in cases where there is difficulty in opening the mouth, as in anchylosis of the jaw, for if vomiting occurred the patient would be liable to asphyxia. One or two deaths from this accident have been reported.

Inquiry should be made in regard to the man's habits, whether he is a drinking man or not. The heart and lungs should be examined. The evidence of weak heart would be a contra-indication to the use of chloroform. It is important that these organs should be examined, for if anything happens to the patient, and you are asked in court if you had examined the heart and lungs, you will be able to say that you had. The presence of cardiac or pulmonary disease is no contra-indication, but in valvular disease of the heart the administration will be made more carefully. In capillary bronchitis I should be chary about administering an anæsthetic, for the amount of mucous secreted may cause asphyxia. In congestion of the brain, if I used ether at all, I should do it very carefully.

Having the patient with an empty stomach, and all constricting clothing removed, and in a recumbent posture, the next thing is the inhaler. The usual inhaler is a towel formed into a cone, with a piece of waxed paper on the outside. Numerous forms of inhalers have been devised. Here is the arrangement of Dr. O. H. Allis, of this city. This answers very well. The temperature of the room should be about 70°. Not too cold, or the ether will not evaporate with sufficient rapidity. If it is too hot, the room will be saturated with the vapor of ether.

Having placed the head in an easy, comfortable position, I lay a towel over the eyes to keep out the sight of surrounding objects. There should be no loud talking in the room. The patient should be assured that there is no danger. This is of especial importance in the case of nervous ladies. They are apt to be frightened, and a frightened patient never takes ether well. In fact, some deaths which have occurred shortly after the beginning of the anæsthesia have been attributed to fright. Some have died before a drop of the anæsthetic was administered The patient must be assured that there is no danger, and should be instructed as to the manner of breathing. He is to take a deep inspiration, and not expel the air too rapidly. This gives more time for absorption. The patient should be allowed to take several long breaths in this manner before the ether is administered. Dr. Bonwill claims that these inspirations produce a species of analgesia. I think that it does obtund the sensibility of the bronchial mucous membrane, and for that reason the ether is received more kindly, and is not so apt to produce coughing.

We next pour one or two drachms of ether on the towel, and hold it to the patient's face. Do not apply it close at first, for this alarms him ; allow him to get some air. For this purpose the bottle devised by Dr. John Roberts does very well. The tube may be inserted in the apex of the cone, and a few drops of ether poured on the towel every few minutes. When the patient begins to come under its influence—which will be in a few minutes—he may become excited, and, perhaps, struggle a little. You can then pour half an ounce of ether on the towel at a time and hold it close to the face, and the patient is soon etherized.

This takes from eight to twenty minutes ; the average is probably about twenty minutes. In old persons and in children it does not take so long, while in healthy adults it requires a longer time. The length of time, however, depends largely on the skill of the anæsthetizer.

Do not use a towel placed flat on the face. I do not see how a patient can get much ether in such a way. If he struggles, and the towel is held on the face, he becomes unconscious ; but this is to a large extent dependent on asphyxia. A cone made in the way described holds a considerable amount of the ether vapor.

It is possible to induce anæsthesia very rapidly. I have rendered a patient unconscious in one minute by dipping a towel in hot water, wringing it out, and sprinkling over it an ounce or two of ether. Dr. Muller, of Germantown, has devised an apparatus for this purpose, in which the ether is vaporized by placing the bottle containing it in a bucket of warm water. The vapor is conducted to the patient through a tube and mask, provided with a stop-cock to regulate the flow of ether. He has etherized a patient in less than a minute. There is less danger from rapid anæsthesia with ether than with chloroform. With the latter agent it should never be attempted One objection to rapid etherization is, that a patient who has taken it once cannot be induced to take it again. I think that it must be a little dangerous, for it does not give a chance to watch the patient, and properly avert any accidents that may arise. I think that the slow method is the safer.

Having gotten the patient under the influence of the anæsthetic, a drachm or two should occasionally be poured on the towel, so as to keep him under it. Do not pour on a large quantity at once, and overwhelm the patient and stop the operation Do not let him come out of the ether, for then he begins struggling. Pour on a small quantity, as I have said, every few minutes, and keep the patient gently asleep. You know that the patient is asleep, from his snoring respiration, and from the insensibility of the conjunctiva. The conjunctiva and the genital organs are the last to lose sensitiveness. If the ether is kept up for some time the face becomes pale. If sick stomach supervenes, the face also becomes pale. Often in the beginning of the administration the face will become pallid, from sick stomach, but if you are sure there is nothing in the stomach, vomiting may be prevented by pushing the ether. If, however, the patient has eaten a hearty meal. it is better to let him vomit.

During the administration of ether, the bronchial mucous membrane secretes a large quantity of. mucous, which may be so large as to almost choke the patient. If this happens, turn the patient on his side, and with the end of a towel remove the mucous. If the mucous is not removed it prevents the patient from inhaling the ether well.

I was called to give ether in one case in which, in previous trials, the patient had secreted mucous so rapidly that he could not be etherized. I overcame the difficulty by administering a glass of whisky and some morphia half an hour before giving ether. Although there was a large quantity of mucous secreted, enough ether was inhaled to render him insensible.

It has been said that some people cannot be anæsthetized. I have never seen such a person. I have been able to anæsthetize every one in whom I have tried to induce this condition, although some required more care than others.

Never allow a patient to be under ether a moment longer than necessary. The condition of shock which it induces tends to retard recovery. Always operate the moment the patient is ready.

There are other ways of administering the ether than those which I have described. It may be administered by the rectum. Dr. Miller, of this city, read a paper before the County Medical Society describing four cases in which he had done this, it might be said, successfully. The ether is vaporized by being placed in water of a temperature of 120°, and the vapor conducted through a tube to the rectum, which has previously been cleared by an injection. Anæsthesia is produced in from six to eight minutes. The abdomen is so distended and there is such a sense of burning that this is scarcely a desirable method. It might be employed, possibly with advantage, in some operations about the mouth. The four cases which were reported by Dr. Miller are the only ones in this city in which this plan has been tried, as far as I know.

After the administration of ether, there is a certain amount of lowering of the body temperature. If the patient is cold and much depressed, the use of hot water bottles will be of advantage. Or a spirit lamp with a tube ending under the bed clothing may be employed.

The sick stomach which often follows the administration of ether is annoying. It is difficult to do much for its relief. The patient may rinse the mouth with hot water, and if he can swallow some of it and vomit he will get rid of considerable ether. The following is sometimes of service:

℞. Spirit. chloroformi........................gtt. viij,

Aceti opii..................................gtt. iij,

Mucilaginis acaciæ, } aa.....................q. s. M.

Aquæ ............ }

This may be repeated every two or three hours.

A sick stomach may often be prevented by administering a hypodermic of morphia before the patient comes out of the ether. It induces sleep until the effects of the ether have entirely passed off.

Before leaving the subject of ether, I wish to call attention to the fact that ether vapor is very inflammable. When operating at night, as you will, see to it that the light is placed higher than the ether, for, as the vapor of ether is heavier than air, it cannot then reach the light. In applying the actual cautery to the face when ether has been administered, all that is necessary to prevent the vapor from catching fire is to remove the cone and pass a fan a few times in front of the patient, thus diluting the vapor.

Mixed anæsthetics are unsafe, on account of the difference in their rate of evaporation, and one cannot judge of the quantity of ether that is being administered. I never use them.

What have I to say of chloroform? As far as this country is concerned, it is rarely used. I used it for a number of years, and never had an accident with it, but I cannot recommend it to you unless you are taught how to use it. My instructor was the late Professor Gross, who never had a death from chloroform. I have given it to children eight days old, and to people eighty years of age.

Although, in some cases, chloroform is better than ether, I cannot advise you to use it. If every hospital had a professional anæsthetizer, he would become proficient in its use. It is said that chloroform does not kill children easily. You should be as careful with children as with adults.

The most dangerous subject for the administration of chloroform is a strong, vigorous man, who, in his struggles, may draw a large quantity of the vapor; spasm of the glottis takes place, and he cannot expel it, and death may follow. Old and weakly people bear chloroform well. I think that there is really less shock from chloroform than from ether. Chloroform has caused many deaths, but it is still successfully used in many country districts. It has been said by a distinguished author, that "the man who persists in the use of chloroform in the face of all the evidence we have against it, is beyond the pale of argument." I rarely use chloroform, except, occasionally, in children, and in some cases of bronchial irritation. I am not afraid to use it, but do not use it because ether answers as well in most cases, and is safer. But do not forget that deaths have occurred from ether; over sixty have been reported, and I am sure many more have occurred of which we shall never hear.

As my hour has expired, I shall, at a future time, have more to say about the administration of chloroform.

